# Location, location, location: Use of CRISPR-Cas9 for genome editing in human pathogenic fungi

**DOI:** 10.1371/journal.ppat.1006209

**Published:** 2017-03-30

**Authors:** Aaron P. Mitchell

**Affiliations:** Department of Biological Sciences, Carnegie Mellon University, Pittsburgh, Pennsylvania, United States of America; McGill University, CANADA

Clustered Regularly Interspaced Short Palindromic Repeat (CRISPR)-Cas9 systems enable the targeting of a double-strand break in genomic DNA to a location chosen by the investigator [1]. The break may be repaired (Fig 1) by cellular nonhomologous end joining (NHEJ) machinery to yield small insertions and deletions [2], which are often loss-of-function mutations in coding regions. If a repair template that spans the break site is available, the break may be repaired by cellular homology-dependent repair (HR) machinery to create a precisely designed insertion, deletion, gene or promoter fusion, or base substitution [2]. CRISPR-Cas9 systems can be programmed to define the location of a break; hence, an investigator can determine the location of a targeted mutation (Fig 1). This technology has revolutionized genetic manipulation of model organisms, cell lines, animals, plants, and even human embryos [1]. The technology has been adapted for use in a variety of fungi (reviewed in [3]), including *Saccharomyces cerevisiae* [4] and multiple *Aspergillus* species of agricultural and industrial importance [5]. Among human fungal pathogens, CRISPR-Cas9 genome editing has been applied successfully to *Aspergillus fumigatus* [6,7], *Cryptococcus neoformans var*. *neoformans* [8], *C*. *neoformans var*. *grubii* [9], *Candida albicans* [10,11], and *Ca*. *glabrata* [12]. In these organisms, CRISPR-Cas9 systems have accelerated the creation of gene-targeted mutations, often without selection for the mutant phenotype, and have enabled the modification of multiple genes or alleles in a single transformation.

**Fig 1:**
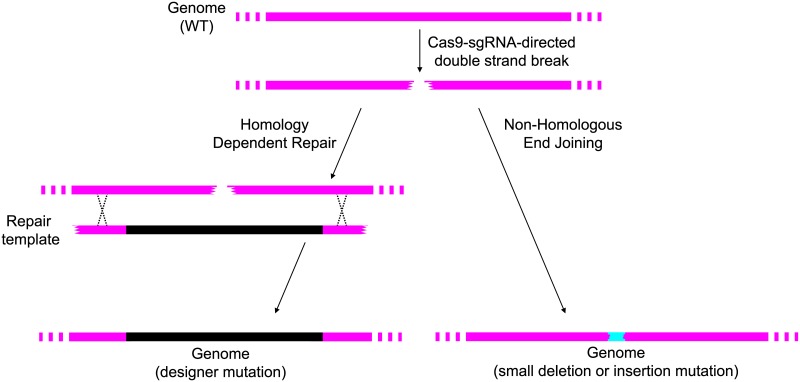
Outcomes of Cas9-single guide RNA (sgRNA) cleavage at a genomic site. The Cas9-sgRNA complex is able to target a double-strand break in DNA to a specific site in the genome. The break is repaired by cellular machinery to generate a mutation that alters the site, thus preventing repeated cleavage. If the cell has a template that has homology to both sides of the break (left side of panel), then the template can be used by homology-dependent repair. For genome engineering, the investigator introduces into cells a custom template along with sources of Cas9 and the relevant sgRNA to create a designer mutation. Such mutations may be small changes in nucleotide sequence or a large insertion or deletion. Alternatively, the cell can use nonhomologous end joining (right side of panel) to create small insertions or deletions at the cut site.

## CRISPR-Cas9 components

The nuclease that creates double-strand breaks has two components: the Cas9 protein and a single guide RNA (sgRNA) [1]. The commonly used *CAS9* gene originated in *Streptococcus pyogenes*, and it has been modified for use in eukaryotic cells through inclusion of gene segments for a nuclear localization sequence and, in many cases, an epitope tag. *CAS9* genes used in most fungi come from versions that were codon-optimized for human cells [6,7,8,9,11,12]; the *CAS9* gene used in *Ca*. *albicans* was modified to accommodate the species' variant genetic code [11]. In all described human fungal pathogen Cas9 systems, the modified *CAS9* gene is expressed constitutively from a fungal RNA polymerase II promoter [6,7,8,9,11,12].

Cas9 associates with the sgRNA, whose sequence directs DNA cleavage through base-pairing with one strand of the target DNA [1]. The sgRNAs that are used in most genome editing systems derive from an engineered fusion of the two natural Cas9-associated RNAs, the CRISPR RNA (crRNA) and the transactivating CRISPR RNA (tracrRNA) [1]. The sgRNA must be uncapped for function. In the described human fungal pathogen systems, the uncapped sgRNA is often expressed from an RNA polymerase III promoter [6,7,8,11,12]. This expression strategy imposes the constraint that the 5′ sgRNA residue must be G, which, in most systems, precedes the targeting region of the sgRNA. If the G is included in the targeting region, this constraint may limit the number of accessible target sites, though it has been argued that a one base-pair mismatch at the 5′ sgRNA residue may have little impact [13]. A second expression strategy was implemented by Arras et al. [9], who took inspiration from plant genome editing systems [14], in which sgRNA is produced via flanking self-cleaving ribozyme sequences after expression from an RNA polymerase II promoter. Finally, it should be noted that in vitro-synthesized sgRNA, included in the transformation mix, has been shown to function in *A*. *fumigatus* [7].

## Introduction of CRISPR-Cas9 components into cells

In many systems, the Cas9 and sgRNA genes are carried on one or two cassettes that are integrated into a genomic site or, if the organism permits, present on plasmids. One generally useful approach is to first create a strain that expresses Cas9, then follow up with a second transformation that introduces an sgRNA gene or in vitro-synthesized sgRNA and, if desired, a repair template [6,7,9,11,12]. It has been demonstrated rigorously that Cas9 expression is innocuous in *A*. *fumigatus*, *Ca*. *albicans*, and *C*. *neoformans* [6,9,11]. A single Cas9-expressing strain can thus be used to create mutations in diverse genes that are determined by the choice of sgRNA and repair template.

The Cas9-sgRNA complex is needed only transiently to create a recombinogenic/mutagenic double-strand break [1]. In fact, there are cases in which Cas9 expression affects growth or virulence [8,12], in contrast to those situations mentioned above. Hence, it may be important to eliminate the source of Cas9 for phenotypic analysis of a newly generated mutant. This problem has been solved by creating situations in which the genes specifying Cas9 and sgRNAs are present only transiently in recipient cells. One solution, applied to *C*. *neoformans* [8], was an elegant modification of the Capecchi homology-directed gene knockout construct for mice [15]. The repair template had appended, at one end, the genes specifying Cas9 and the sgRNA [8]. The idea was that homologous recombination at both sides of the genomic cut site would yield an isolated linear DNA segment specifying Cas9 and the sgRNA, which would be degraded and lost [8]. A second solution, applied to *C*. *albicans*, came from the observation that many Cas9-sgRNA—directed deletion/insertion mutants did not carry integrated copies of the genes specifying the sgRNA and Cas9 [10]. In this transient system, linear PCR products corresponding to the genes for the sgRNA and Cas9, and lacking a selection marker, were cotransformed into cells along with the selectable repair template [10]. The linear PCR products apparently functioned, as indicated by the frequency of mutant recovery, but were not maintained in the genome [10]. These transient systems may become popular for fungal pathogen genome engineering because of their simplicity, requiring only PCR and no cloning.

## On the near horizon

Some of the most burning questions in the field of genome editing have to do with off-target effects [1,16]: How can off-target effects be assessed? How can they be minimized? Many off-target sites in other systems have one or a few mismatches to the sgRNA, particularly in its 5′ region, so mutations in candidate off-target sites may be tested through sequencing or nuclease-based assays [16]. The capture of break sites for sequencing or whole-genome sequencing are also useful approaches [16]. Technologies to minimize off-target effects are now maturing. For example, the use of paired Cas9 mutants that nick only a single strand can improve specificity considerably [17], and high-specificity Cas9 variants have been engineered recently [18]. Alternatively, use of 5′-truncated sgRNAs has been shown to improve cleavage site specificity [19]. It is encouraging that a 5′-truncated sgRNA has been shown to function for on-target effects in *A*. *fumigatus* [7]. However, there have been few assessments of off-target effects in the human fungal pathogens. Interestingly, this field has a long-standing tradition of concern about secondary mutations and their phenotypic impact. Investigators have addressed this concern through analysis of complemented derivatives of mutant strains. Thus far, only one Cas9-sgRNA—directed mutation in a human fungal pathogen has been subjected to validation through complementation [8]. To be fair, the test loci used for development of Cas9-sgRNA systems have been well characterized previously, and the newly created Cas9-sgRNA—directed mutations generally have the expected phenotypes. However, the development of facile complementation or gene-reconstitution systems to accompany genome editing technology will be critical to address newly accessible questions about gene functions and interactions.

There is also a long-standing question in the fungal pathogen field that is now more accessible than ever: how universal are the virulence and drug resistance determinants defined through genetic manipulation? Most gene function studies thus far have focused on one or two isolates of a pathogen that are amenable to genetic analysis. Yet there are many examples in which detailed analysis of a new clinical isolate can break ground in our broader understanding of pathogen biology and drug resistance [20,21,22,23]. Perhaps the most important lesson from the genome editing field is that the breadth of strains and species that can be subjected to targeted genome modification is nothing short of incredible! One exciting opportunity on the horizon, illustrated in the study of Vyas et al. [11], is to validate prospective drug targets and explore pathogenesis mechanisms in a range of clinical isolates of the major human fungal pathogens.
